# The Effect of Stretching on Ultraviolet Protection of Cotton and Cotton/Coolmax-Blended Weft Knitted Fabric in a Dry State

**DOI:** 10.3390/ma6114985

**Published:** 2013-10-31

**Authors:** Chi-wai Kan, Lim-yung Yam, Sun-pui Ng

**Affiliations:** 1 Institute of Textiles and Clothing, The Hong Kong Polytechnic University, Hung Hom, Kowloon, Hong Kong 852, China; E-Mail: eddieyly@yahoo.com.hk; 2 Hong Kong Community College, The Hong Kong Polytechnic University, Hung Hom, Kowloon, Hong Kong 852, China; E-Mail: ccspng@hkcc-polyu.edu.hk

**Keywords:** cotton, coolmax, weft knitted fabric, ultraviolet protection factor (UPF), dry, relax, stretching

## Abstract

In this paper, the ultraviolet protection factor (UPF) of weft knitted fabrics made from 20Ne cotton yarn, Coolmax yarn and their blends in dry, relaxed and stretched states were studied. According to the fibre composition, samples were divided into three groups: Group I (single cotton yarn); Group II (cotton/cotton combination); and Group III (Coolmax/cotton combination) for discussion. In addition, yarn and fabric properties such as yarn tenacity, yarn strength, fibre combination and water vapour transmission that affect the corresponding UPF values are used for formulating a prediction model in order to determine UPF. Generally speaking, when samples are measured under stretched conditions in a dry state, they exhibit a remarkable reduction in ultraviolet protective power, as pores are opened up and UV radiation can easily penetrate through these pores. In addition, greater stretch percentage came along with greater reduction in UPF. This can be explained by the fact that the amount and the size of pores increase when samples are subjected to greater tension.

## 1. Introduction

Knitwear prevails as an indispensable type of garment for the summer. The UV protection provided by a regular single jersey white garment can be good if measured in the relaxed state. Osterwalder *et al.* [1] carried out a study for quantifying the change in UPF when stretched. It was demonstrated that the change of UV radiation transmittance spectrum *T_λ,x_* can be modelled with a Lambert–Beer type of approach as shown in Equation (1) [2].

(1) $$T_{\text{λ,}x}=P_{x}+(1-P_{x})\times 10^{-A_{\text{λ}}d_{x}}$$

In Equation (1), optical porosity *P_X_* was first measured at various degrees of stretch. The absorption of the fabric is the product of the absorption coefficient *A_λ_* and the optically effective layer thickness *d_x_*. The thickness is reduced by stretching, according to the principle of volume retention of the fabric and *x* is the magnitude of stretch. UV radiation penetration increases almost linearly with stretch. By using this equation, the increase in UV radiation penetration (*i.e.*, reduction in UPF) can be predicted from the transmittance spectrum in the relaxed state. 

Several studies have suggested that fabric under stretched conditions generally has a lower UPF [1,3,4,5,6]. The rationale is that the pores in the fabric structure are widened when it is stretched. Clark *et al*. [3] found out that UPF rating of a cotton/Lycra knitted fabric decreases from 23 to 10 with a course-wise stretch of 15% and drop from 23 to 14 under the same stretch conditions in wale-wise direction. Moreover, Kimlin *et al.* [7] also determined that the original UPF of 50 of a stocking can decline as much as nine times after a 30% stretch in both lengthwise and cross-machine directions.

In daily activities, human sweat and moisture must be evaporated from the skin. Coolmax is a modified polyester fibre with cross-sectioning of the proprietary tetra channel. This feature has the ability to pull moisture from the skin in a process called “wicking.” To be more specific, the Coolmax fabric absorbs and spreads moisture out across fabric thickness to enhance evaporative drying as this increases surface area by 20% compared with yarn of the same linear density. During the wicking process, air is moved in to keep the body dry and cool. Cotton, on the other hand, absorbs and retains 14 times more moisture than Coolmax. The engineered inherence moisture management properties thus make it suitable for lightweight summer clothing. In this paper, therefore, the influence of stretching on the UV protection of cotton and cotton/Coolmax-blended weft knitted fabric in a dry state is studied.

## 2. Experimental Section 

### 2.1. Yarn Information

Grey cotton yarns and Coolmax yarn were supplied by The Central Textiles (H.K.) Ltd. (Hong Kong, China) and Shanghai Ming Mao Industrial Co., Ltd. (Shanghai, China), respectively. The yarn information is shown in Table 1. In order to study the influence of different yarn combinations, three types of yarn were studied: Group I (single cotton yarn); Group II (cotton/cotton combination); and Group III (Coolmax/cotton combination), with combination details as shown in Table 2. Torque-free ring spinning is a technique for producing yarn with a torque-reduction device in the conventional ring-spinning system that modifies the yarn structure [8,9,10,11].

**Table 1:** Yarn specification.

Code	Fibre Type	Spinning Method	Twist Number per 1 cm	Yarn Count
CH	Combed Cotton	Conventional Ring Spun	6.92	Ne 20
MCG	Combed Cotton	Torque-Free Ring Spun	4.68	Ne 20
F	Combed Supima Cotton	Conventional Ring Spun	5.38	Ne 20
MF	Combed Supima Cotton	Torque-Free Ring Spun	4.20	Ne 20
CM	Coolmax	Filament	1.03	150 dtex

**Table 2:** Yarn combinations.

Group	Code	Fibre Type in Yarn Combination	Spinning Method
Group 1	CH	Combed Cotton	Conventional Ring Spun
	MCG	Combed Cotton	Torque-Free Ring Spun
	F	Combed Supima Cotton	Conventional Ring Spun
	MF	Combed Supima Cotton	Torque-Free Ring Spun
Group 2	CH-MCG	Combed Cotton + Combed Cotton	Conventional Ring Spun + Torque-Free Ring Spun
	CH-F	Combed Cotton + Combed Supima Cotton	Conventional Ring Spun + Conventional Ring Spun
	CH-MF	Combed Cotton + Combed Supima Cotton	Ring Spun + Torque-Free Ring Spun
	MCG-F	Combed Cotton + Combed Supima Cotton	Torque-Free Ring Spun + Conventional Ring Spun
	MCG-MF	Combed Cotton + Combed Supima Cotton	Torque-Free Ring Spun + Torque-Free Ring Spun
	F-MF	Combed Supima Cotton + Combed Supima Cotton	Conventional Ring Spun + Torque-Free Ring Spun
Group 3	CM	Coolmax	Filament
	CM-CH	Coolmax + Combed Cotton	Filament + Conventional Ring Spun
	CM-MCG	Coolmax + Combed Cotton	Filament + Torque-Free Ring Spun
	CM-F	Coolmax + Combed Supima Cotton	Filament + Conventional Ring Spun
	CM-MF	Coolmax + Combed Supima Cotton	Filament + Torque-Free Ring Spun

### 2.2. Weft Knitted Fabric Preparation

Based on the yarn combinations given in Table 2, 15 types of plain knitted fabrics were produced from a DXC single jersey machine (Fukuhra, Japan). The knitting machine was 18 inches in diameter, with 54 feeders and 20 gauges with 2 cam tracks selection. Fabric samples were divided into three groups for study, as in Table 2. The combined scouring and bleaching process was carried out as pretreatment and the treatment bath, containing Sandopan DTC (5 g/L), sodium hydroxide (10 g/L), Stabilizer AWN (1 mL/L) and 35% hydrogen peroxide (25 mL/L), was prepared. Fabric samples were padded with the liquor at 30 ^○^C until 100% wet pickup. Those padded fabric samples were steamed for 30 min at 102–105^○^C and then were rinsed thoroughly in hot and cold water. Finally, the fabric samples were laid flat and air-dried completely in the conditioning room with a relative humidity of 65% ± 2% and a temperature of 20 ± 2^○^C in order to avoid shrinkage during drying. After drying, the fabric samples were conditioned at a relative humidity of 65% ± 2% and a temperature of 20 ± 2^○^C for at least 24 hours before use.

### 2.3. Yarn Properties Measurement

All yarn (in cones) was conditioned with relative humidity of 65% ± 2% and temperature of 20 ± 2^○^C for at least 24 hours before use. Yarn strength and tenacity was measured by USTER TENSORAPID 4, and unevenness was measured by USTER evenness tester.

### 2.4. Water Vapour Transmission

Water vapour transmission (WVT) of knitted fabric samples was measured in accordance with ASTM E96-2010. 

### 2.5. Ultraviolet Protection Factor Evaluations

#### 2.5.1. Evaluations under Dry and Relaxed Conditions

Fabric samples were conditioned at a relative humidity of 65% ± 2% and a temperature of 20 ± 2^○^C for 24 hours before UPF measurements by Cary-300 with Lapsphere were made. The AS/NZS 4399:1996 was used and the UPF rating was derived from Equation (2). Readings of each fabric sample were taken from four positions and four times at each position (rotate 90^○^ clockwise after each measurement) and afterwards the average UPF values were calculated from the readings.

(2) $$\text{UPF}=\frac{\sum_{290}^{400}E_{\lambda }\times S_{\lambda }\times \Delta \lambda }{\sum_{290}^{400}E_{\lambda }\times S_{\lambda }\times T_{\lambda }\times \Delta \lambda }$$

where *E_λ_* is relative erythemal spectral effectiveness; *S_λ_* is solar spectral irradiance in Wm^−2^nm^−1^; *T_λ_* is spectral transmittance of the item; *∆λ* is wavelength step in nm and *λ* is wavelength in nm.

#### 2.5.2. Evaluations under Dry and Stretched Conditions

**Figure 1:**
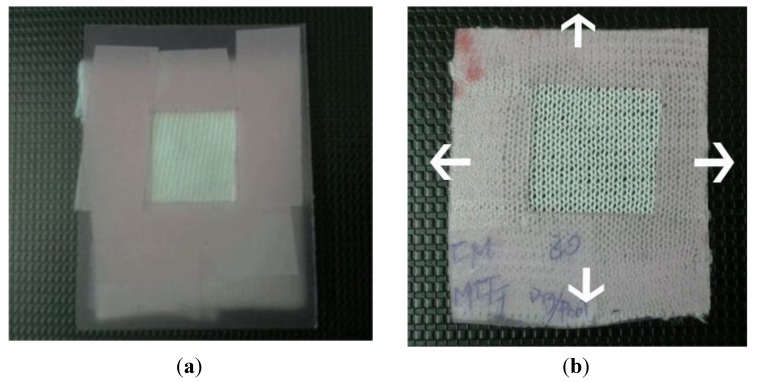
(**a**) Relaxed samples; and (**b**) stretching of samples at 30% in both lengthwise and cross-machine directions (e.g., CM_MCG).

Stretching is another factor that may affect the UPF. Fabric samples were stretched in both lengthwise and cross-machine directions to three levels, *i.e.*, 10%, 20% and 30% of the original dimension (Figure 1 shows an example of stretching). 

### 2.6. Microscopy

Leica M125 stereomicroscope was used for viewing the pore sizes of fabric samples under different stretch conditions at 5 × magnification. Single yarn was viewed at 12.5 × magnification.

### 2.7. Determination of the Size of Pores on Samples under Stretching

Image-processing software, Photoshop CS5, was used to determine the ratio of the size of pores to the whole fabric sample. In this evaluation, fabric sample images under different stretching conditions were captured by a digital camera. This test simply selected the pixels that emerged under stretching and computed its ratio to the whole picture and finally expressed in terms of percentage. 

### 2.8. Prediction Model of UPF

SPSS was used to develop a prediction model by means of multiple linear regressions (MLR) for UPF under different testing conditions. In this model, the dependent variable was UPF and the independent variables were tenacity, yarn strength, fibre combination and water vapour transmission (WVT). A stepwise regression analysis with a confidence level of 95% was used in the current study. Hence, variables with *p*-values less than or equal to 0.05 were included in the final model, whereas those with *p*-values greater than 0.05 were omitted. The significance of the prediction model was also evaluated by different tests in SPSS.

## 3. Results and Discussion

**Figure 2:**
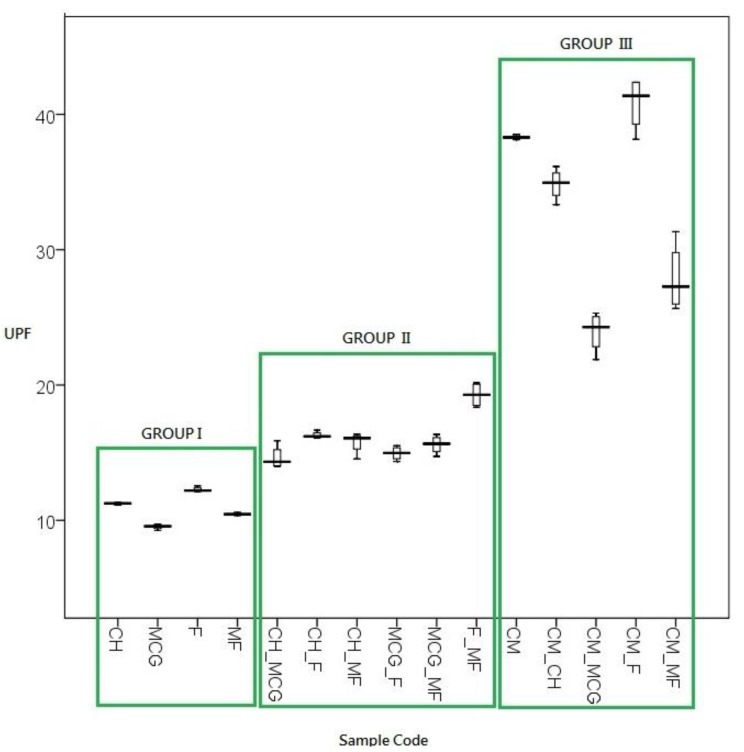
Box-and-whisker plot of UPF results of fabric samples in the three groups at dry and relaxed states.

Figure 2 shows the box-and-whisker plot of UPF results of the three groups of fabric samples in dry and relaxed states. In this plot, the middle horizontal line of each box represents the median value of UPF. The top and bottom edges of the box are the upper and lower quartile lines, respectively, while the end points of the whiskers are the extreme UPF values. The results are discussed in detail as follows. 

### 3.1. Group I (Single Cotton)—UPF at Dry and Relaxed States

The average UPF value is 11.24 for Group I (CH, MCG, F and MF), *i.e.*, fabric samples made by a single yarn. In addition, variations in UPF values of each sample were smaller when compared with the other two groups, as shown in Figure 2. 

#### 3.1.1. Comparison of Spinning Methods

Conventional ring-spun yarn sample CH provides a better UPF rating than torque-free ring-spun yarn sample MCG, provided the fibre type is the same. Thus, the spinning method of yarn affects the UPF. The fibre types of CH and MCG are the same and only differ in spinning method; the same is observed in a comparison of samples F and MF. Both fabric samples F and MF are produced by the same cotton fibre (combed Supima cotton) and only the spinning methods are different. Fabric sample F is produced from conventional ring-spun yarn while fabric sample MF is produced from torque-free ring-spun yarn. In this study, conventional ring-spun yarn can provide 15.21% better UPF than torque-free ring-spun yarn [UPF: CH (11.24) > MCG (9.53)] in the case of combed cotton. For the combed Supima cotton, conventional ring-spun yarn can provide 14.75% higher UPF than torque-free ring-spun yarn [UPF: F (12.27) > MF (10.46)]. 

Torque-free ring spinning produces yarn with a torque reduction device in the conventional ring-spinning system and the yarn structure is modified [8,9,10,11]. Yarn twist can be reduced to a great extent [8,9,10,11]. Less twist of torque-free spun yarn (Table 1) complies with previous findings [8,9,10,11]. Twists per one centimeter of CH = 6.92 > MCG = 4.68 and F = 5.38 > MF = 4.2, *i.e.*, conventional ring spun > torque-free ring spun. A higher twist results in an uneven yarn surface and thus facilitates reflection and scattering of UV radiation [12]. 

#### 3.1.2. Comparison of Fibre Types

Combed Supima cotton yarn “F” provides a better UPF rating than combed cotton yarn “CH” when using the same spinning method, thereby implying that fibre type affects UPF of knitted fabrics. The spinning methods of these two samples are the same and differ only in fibre content; the same is observed in a comparison of MF and MCG. Both F and CH were produced by the same method (conventional ring spinning). The fabric sample “F” is made from combed Supima cotton yarn while fabric sample “CH” is made from combed cotton fibre. Combed Supima cotton fibre is commonly known as an extra long staple. According to Cotton Incorporated, if the upper half mean (UHM) length of upland fibre is longer than 32 mm, then it is categorized as an extra long staple (Cotton Incorporated and Textile World, 2003). Combed Supima cotton yarn can provide 8.36% better UPF than combed cotton fibre yarn [UPF: F (12.27) > CH (11.24)] in the case of conventional ring spinning. Combed Supima cotton yarn can provide 8.88% better UPF than normal cotton fibre yarn [UPF: MF (10.46) > MCG (9.53)] in torque-free ring spinning.

Surface unevenness of combed Supima cotton yarn is lower than that of combed cotton yarn as shown in Table 3. Smoother combed Supima cotton yarn can produce higher uniformity fabric surface. In addition, the torque-free ring-spun yarn can improve textiles surface roughness by about 12%–17% [9], which can thus give a better reflection of UV radiation than fabric produced from a conventional ring-spinning process.

**Table 3:** Surface unevenness of cotton yarn samples.

Sample Code	Yarn Surface Unevenness	Fibre Type
CH	8.43	Combed Cotton
MCG	8.23	Combed Cotton
F	6.98	Combed Supima Cotton
MF	7.13	Combed Supima Cotton

### 3.2. Group II (Cotton/Cotton Combinations)—UPF at Dry and Relax State

The average UPF rating is 14.89 for Group II, which is 25% better than Group I, as shown in Figure 2. Fabric sample F_MF shows the best UPF among this group. Two combed Supima cotton yarns blended together bring out better UV radiation protective power than combed cotton blended with combed Supima cotton. The worst combination is combed cotton blended with combed cotton, *i.e*., sample CH combined with sample MCG. The effect of fibre type outweighs the effect of spinning type in this group. 

The other observation is the overall variations of the ratings increase compared with that for Group I. When two different yarns are used for knitting at the same time, variation in UPF increases accordingly. As the twist of each type of cotton yarn was not the same, the fabric surface is uneven [12]. This explains the reason of greater variation of UPF in Group II (cotton/cotton combinations). 

### 3.3. Group III (Coolmax/Cotton Combinations)—UPF at Dry and Relax State

**Figure 3:**
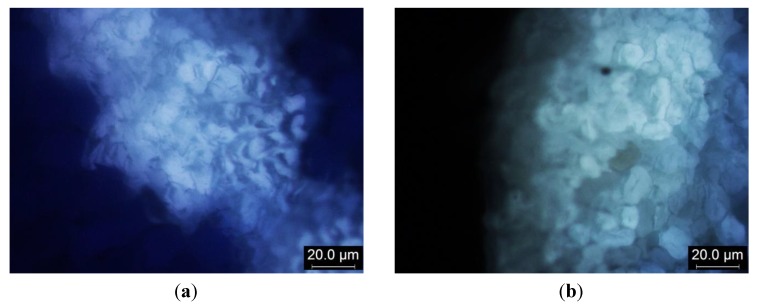
Fibre cross-sectional view of (**a**) Coolmax; and (**b**) cotton.

The average UPF value of Group III is 31.87 (Figure 2), which is 49.20% and 65.86% higher than for Group II and Group I, respectively. Coolmax (CM) provides average UPF rating of 38.32. Coolmax is a kind of polyester that has a different fibre cross-sectional view (Figure 3). The delustrant applied during manufacturing acts as a good UV absorber, so it inherently provides better protection against UV radiation than cotton yarns [13,14]. In addition, the reason for the Coolmax sample yielding a higher UPF than the cotton sample is that Coolmax contains benzene rings and a conjugated aromatic system of polymer chains that are more effective in UV radiation absorption [13,15,16]. On the contrary, cotton (a kind of cellulose) has no double bonds in its chemical structure and thus has a low intrinsic UV absorption capacity and can only provide relatively low UV protection properties than Coolmax [13,15,16]. The results observed in Group III seem to resemble the results of Group I (single cotton) but the values are three to four times higher, which is consistent with Davies *et al.* [16]. The large extent of variations can be explained by the fact that a twist of Coolmax yarn is extremely low, only 1.03 per centimetre (Table 1). 

### 3.4. Prediction of UPF at Dry and Relaxed Statse (UPF_dry and relaxed_)

The UPF of fabric varies linearly with fibre properties. In the present investigation, yarn tenacity at break (tenacity), yarn strength, fibre combination and water vapour transmission (WVT) were used to formulate a prediction model of UPF at dry and relaxed states (UPF_dry and relaxed_) by MLR. Equation (3) shows the proposed prediction model for UPF_dry and relaxed_.

*Y = a + b*_1_(*X*_1_) *+ b*_2_(*X*_2_) *+ b*_3_(*X*_3_) *+ b*_4_(*X*_4_) (3)

where *Y* is the UPF of dry and relaxed plain knitted fabric (UPF _dry_ _and_ _relax_); *X*_1_ is yarn tenacity (cN/tex); *X*_2_ is yarn strength (N); *X*_3_ is fibre combination (1: cellulose fibre, 2: cellulose combination, 3: synthetic fibre and 4: cellulose/synthetic combination) and *X*_4_ is water vapour transmission (WVT); *b_i_* (*i* = 1, 2, 3 and 4) is the related regression coefficient of *X_i_* (*i* = 1, 2, 3 and 4) and *a* is the intercept of Equation (3).

By computing the relevant information using SPSS, values of *a*, *b*_1_, *b*_2_, *b*_3_ and *b*_4_ can be found in Table 4 while the prediction model for UPF_dry and relaxed_ is shown in Equation (4). From this equation, it is observed that WVT affects the UPF most, whereas the other parameters have little effect. The coefficient of multiple determination (*R*^2^) was found to be 0.907. This value indicates that 90.7% of the variation in the UPF_dry and relaxed_ can be explained by variables of yarn tenacity, yarn strength, fibre combination and water vapour transmission.

**Table 4:** Coefficient table for model predicting UPF_dry and relax_.

Intercept/coefficient	Value	Significant (Sig).
*a*	13.482	0.05
*b_1_*	1.276	0.00
*b_2_*	–2.129	0.00
*b_3_*	2.900	0.00
*b_4_*	–7.850	0.00

*Y* = 13.482 + (1.276) *X*_1_ + (–2.129) *X*_2_ + (2.900) *X*_3_ + (−7.850) *X*_4_ (4)

### 3.5. Verification of the Model Predictive Ability for UPF_dry and relaxed_

The UPF_dry and relaxed_ can be predicted by using yarn tenacity, yarn strength, fibre combination and water vapour transmission. In order to test how precisely the model can be applied for prediction, verification of the model is needed and results are shown in Table 5 below. 

**Table 5:** Difference (%) between “Actual” and “Predicted” values of UPF_dry_ _and_ _relaxed_.

Group No.	Sample code	UPF		Differences (%) between “Actual” and “Predicted” UPF
		Predicted	Actual	
Group I	CH	11.66	11.24	+3.73%
	MCG	9.44	9.53	−0.99%
	F	13.54	12.27	+9.37%
	MF	11.95	10.46	+12.44%
Group II	CH_MCG	14.44	14.89	−3.14%
	CH_F	15.17	16.29	−7.40%
	CH_MF	16.51	15.76	+4.55%
	MCG_F	16.23	14.96	+7.83%
	MCG_MF	14.04	15.96	−13.66%
	F_MF	17.18	19.27	−12.14%
Group III	CM	37.66	38.32	−1.77%
	CM_CH	33.93	34.84	−2.69%
	CM_MCG	25.96	23.94	+7.76%
	CM_F	37.93	40.82	−7.63%
	CM_MF	31.53	27.88	+11.57%
				Average: 0.52%

Generally speaking, the prediction model for UPF_dry and relaxed_ tends to have good prediction of UPF_dry_ _and_ _relaxed_; the overall difference between all samples is 0.52%. The worst prediction was −13.66% on sample MCG_MF, while the best prediction was −0.99% on sample MCG. There are eleven fabric samples that have differences between actual and predicted UPF values within 10% variation. The coefficient of determination (*R*^2^) of the model is 0.907, which means it can explain 90.7% of the total variance by variables of fibre combination, yarn tenacity, yarn strength and water vapour transmission. The prediction model can thus be concluded as an effective way of predicting UPF_dry_ _and_ _relaxed_ state even for blended fibre combinations. 

### 3.6. UPF at Dry and Stretch State

**Figure 4:**
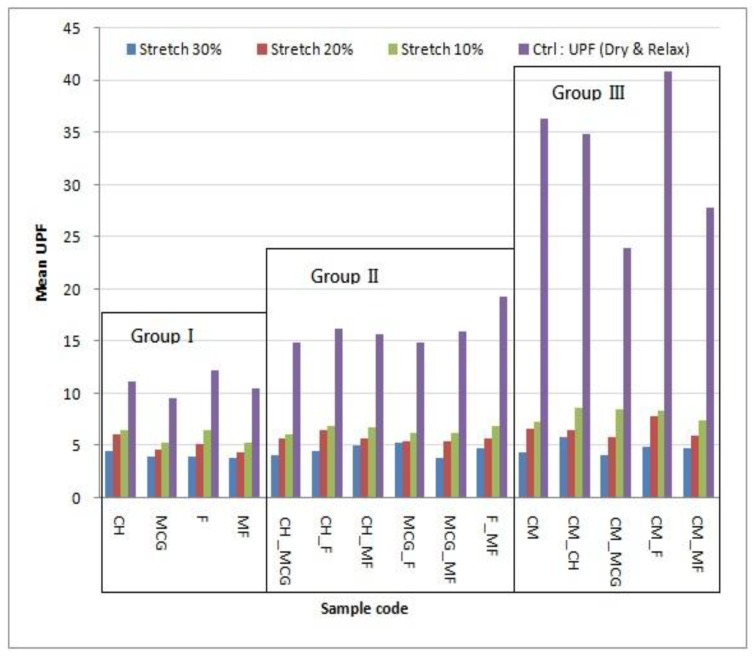
Overall performance on UPF under 3 different stretch conditions.

Fabric structure becomes looser under stretched conditions as the pores between loops are opened up under stretching. UPF values decrease along with an increase in stretch, as shown in Figure 4. 

**Figure 5:**
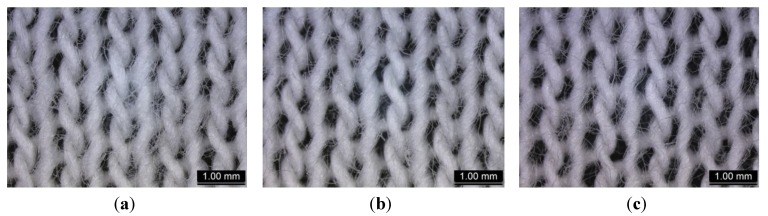
Sizes of holes on sample “CH_F” subjected to (**a**) 10%; (**b**) 20%; and (**c**) 30% stretching in both lengthwise and cross-machine directions.

The pores are larger as the fabric is stretched more, as shown in Figure 5 (using “CH_F” as an example). The magnitude of decrease in UPF is most profound in Group III, compared with Group II and Group I, as shown in Figure 4. As the presence of holes dominates the cause of decreasing UPF, effects of fibre type and the spinning method become insignificant and negligible in this Group.

**Figure 6:**
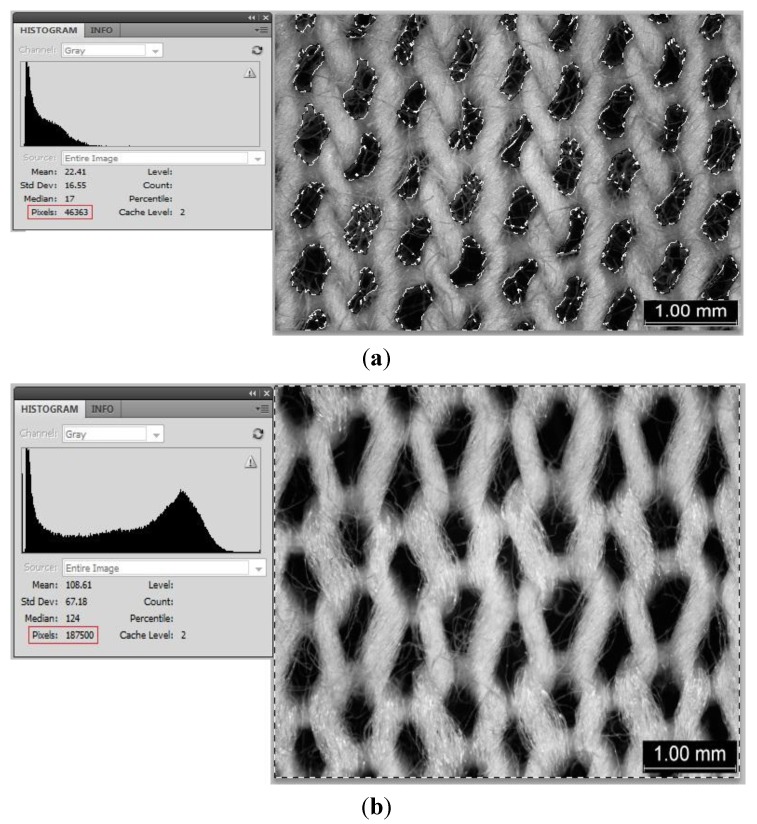
Number of pixels of (**a**) pores; and (**b**) whole picture.

In analysing the size of holes in the fabric, the size of holes can be interpreted as the “black pixels” and it is compared to the whole picture pixels by Photoshop software to determine the open area-to-sample ratio. The black pixels (which are in fact the pores and holes in-between loops emerge under stretching) are selected and determined by Photoshop as shown in Figure 6a. In Figure 6a, the count for total number of black areas is 46,363 pixels. On the other hand, the count for whole picture pixels (including both black and white “areas”) is 187,500 pixels (Figure 6b). Then the black-to-whole picture pixels ratio is 46,363 over 187,500 pixels that equals to 24.73% (CH_F stretch 30%). Table 6 shows that as the stretch increases, the black pixel ratio increases accordingly, which indicates that the stretching could affect the size of holes in the weft knitted fabrics. 

**Table 6:** Black pixel to whole picture’s pixel ratio.

Group No.	Sample code	Black pixel to whole picture’s pixel ratio		
		Stretch 10%	Stretch 20%	Stretch 30%
Group I	CH	8.87%	20.39%	26.11%
	MCG	10.63%	22.16%	23.25%
	F	9.57%	21.47%	24.99%
	MF	10.14%	20.96%	23.19%
Group II	CH_MCG	9.95%	20.41%	23.83%
	CH_F	9.10%	17.57%	24.73%
	CH_MF	7.19%	16.29%	25.04%
	MCG_F	6.71%	17.37%	26.13%
	MCG_MF	10.41%	20.24%	25.91%
	F_MF	8.82%	18.25%	24.36%
Group III	CM	9.26%	17.56%	23.62%
	CM_CH	5.47%	18.50%	21.47%
	CM_MCG	9.94%	18.78%	22.08%
	CM_F	7.64%	20.19%	22.41%
	CM_MF	8.47%	18.65%	23.08%

### 3.7. Prediction of UPF at Dry and Stretched States (UPF_dry and stretched_)

UPF of fabric samples stretched 10%, 20% and 30% are averaged to derive an average value. It is difficult to determine the stretching percentage on a particular part of the clothing during wearing, so the three stretching percentages are averaged to get the general value to become the dependent variable for prediction. In this prediction, yarn tenacity at break (tenacity), yarn strength, fibre combination and water vapour transmission are used to compute and formulate the prediction model of UPF at dry and stretched state (UPF_dry and stretch_) by Multiple Linear Regression (MLR). Equation (3) is used again for the proposed prediction model for UPF_dry and stretched_. By computing the relevant information using SPSS, values of a, b_1_, b_2_, b_3_ and b_4_ can be derived, as shown in Table 7 (coefficient table). As a result, the Multiple Regression Model for UPF_dry and stretched_ is shown in Equation (5).

*Y* = 6.735 + (−0.053) *X*_1_ + (0.216) *X*_2_ + (0.610) *X*_3_ + (−1.125) *X*_4_ (5)

**Table 7:** Coefficient table for model predicting UPF_dry and stretched_.

Intercept/coefficient	Value	Significant (Sig.)
*a*	6.735	0.00
*b*_1_	−0.053	0.01
*b*_2_	0.216	0.04
*b*_3_	0.610	0.00
*b*_4_	−1.125	0.00

### 3.8. Verification of the Model Predictive Ability of UPF_dry and stretched_

The UPF_dry_ _and_ _stretched_ can be predicted by using yarn tenacity, yarn strength, fibre combination and water vapour transmission. In order to test how precisely the regression can be applied for prediction, verification of the regression is needed; results are shown in Table 8.

**Table 8:** Difference (%) between “Actual” and “Predicted” values of UPF_dry_ _and_ _stretched_.

Group No.	Sample code	UPF		Differences (%) between “Actual” and “Predicted” UPF
		Predicted	Actual	
Group I	CH	4.99	5.75	−13.12%
	MCG	4.94	4.66	+6.04%
	F	4.57	5.24	−14.80%
	MF	4.92	4.54	+7.78%
Group II	CH_MCG	5.56	5.28	+5.06%
	CH_F	5.24	5.94	−13.35%
	CH_MF	5.46	5.84	−6.96%
	MCG_F	5.54	5.65	−2.06%
	MCG_MF	5.38	5.22	+2.94%
	F_MF	5.42	5.81	−7.28%
Group III	CM	5.39	5.60	−3.78%
	CM_CH	6.88	7.06	−2.67%
	CM_MCG	6.30	6.15	+2.34%
	CM_F	6.89	7.06	−2.50%
	CM_MF	6.55	6.09	+7.07%
				Average: −2.35%

Generally speaking, the prediction model tends to yield good estimations of UPF_dry_ _and_ _stretched_ and the overall differences among samples are −2.35%. The worst prediction is −14.80% on sample F, while the best prediction is −2.06% on sample MCG_F. In twelve samples, the variation between actual and predicted UPF values was within 10% and the coefficient of determination (*R*^2^) of the model is 0.847 which means the model can explain 84.7% of total variances by variables of fibre combination, yarn tenacity, yarn strength and water vapour transmission. The prediction model can be concluded as a successful way of predicting UPF_dry_ _and_ _stretch_ state even for different fibre combinations.

## 4. Conclusions 

In this paper, UV properties of weft knitted fabric in dry, relaxed and stretched states were studied. The weft knitted fabrics used were divided into three groups, and their results are summarized as follows:

(i) For Group I (single cotton), UPF ratings were compared in two aspects, *i.e.*, spinning method and fibre type. Cotton yarns produced from a conventional ring-spinning method can well protect against UV radiation compared with torque-free ring-spun yarn, and it is believed that the yarn twist plays an important role in affecting UPF. As the yarn twist is directly influenced by the spinning method, it can be concluded that the spinning method can affect UPF. When comparison was done based on the fibre type, combed Supima cotton yarn was found to provide better UPF than combed cotton yarn, with the spinning method being the same. This can be explained by the better reflectance of combed Supima cotton fabric. In short, both fibre type and spinning method affect UPF of fabrics;
(ii) Results for Group II (cotton/cotton combinations) suggest that a combination of two combed Supima cotton yarns provides better UPF than either two combed cotton yarns or one combed cotton yarn combined with a combed Supima cotton yarn. The spinning method became less important in affecting UPF in this group;
(iii) Group III (Coolmax/cotton combinations) had the highest UPF values among the three groups. The results of each blended sample were similar to the samples in Group I but at a higher level, as the presence of Coolmax increases the protective ability against UV radiation.

When samples were measured under stretched conditions in dry state, a remarkable reduction in protective power was observed, as pores were opened up and UV radiation could easily penetrate through the pores. In addition, greater stretch percentages came along with greater reduction in UPF, which can be explained by the fact that the number and the size of pores increase when samples are subjected to greater tension. 
